# Sustainable remediation: electrochemically assisted microbial dechlorination of tetrachloroethene-contaminated groundwater

**DOI:** 10.1111/1751-7915.12089

**Published:** 2013-10-01

**Authors:** Sayali S Patil, Eric M Adetutu, Jacqueline Rochow, James G Mitchell, Andrew S Ball

**Affiliations:** 1 School of Biological Sciences, Flinders University of South AustraliaAdelaide, SA, 5042, Australia; 2 Department of Applied Biosciences, Royal Melbourne Institute of TechnologyBundoora, Vic., 3083, Australia

## Abstract

Microbial electric systems (MESs) hold significant promise for the sustainable remediation of chlorinated solvents such as tetrachlorethene (perchloroethylene, PCE). Although the bio-electrochemical potential of some specific bacterial species such as Dehalcoccoides and Geobacteraceae have been exploited, this ability in other undefined microorganisms has not been extensively assessed. Hence, the focus of this study was to investigate indigenous and potentially bio-electrochemically active microorganisms in PCE-contaminated groundwater. Lab-scale MESs were fed with acetate and carbon electrode/PCE as electron donors and acceptors, respectively, under biostimulation (BS) and BS-bioaugmentation (BS-BA) regimes. Molecular analysis of the indigenous groundwater community identified mainly *Spirochaetes*, *Firmicutes*, *Bacteroidetes*, and γ and δ*-Proteobacteria*. Environmental scanning electron photomicrographs of the anode surfaces showed extensive indigenous microbial colonization under both regimes. This colonization and BS resulted in 100% dechlorination in both treatments with complete dechlorination occurring 4 weeks earlier in BS-BA samples and up to 11.5 μA of current being generated. The indigenous non-*Dehalococcoides* community was found to contribute significantly to electron transfer with ∼61% of the current generated due to their activities. This study therefore shows the potential of the indigenous non-*Dehalococcoides* bacterial community in bio-electrochemically reducing PCE that could prove to be a cost-effective and sustainable bioremediation practice.

## Introduction

Chlorinated ethenes such as tetrachloroethene (perchloroethylene, PCE), trichloroethene (TCE) and dichloroethene (DCE) are among the most frequently detected groundwater pollutants (Moran *et al*., 2007), posing a serious threat to the environment and human well-being because of their carcinogenic properties (ATSDR, 2007). The current *in situ* and onsite bioreactor-engineered approaches for the bioremediation of chlorinated contaminants typically involve the addition of molecular hydrogen (H_2_) or H_2_-releasing organic substrates to stimulate the metabolism of reductive dechlorinating microorganisms. This stimulation facilitates the reduction of PCE to environmentally benign ethene. The problems often associated with this approach include the extensive competition for carbon and H_2_ between dechlorinators and non-dechlorinating sulphate reducers, methanogens and homoacetogens, and accumulation of large amounts of fermentation products in the subsurface. These problems can result in deterioration of groundwater quality, possible aquifer clogging because of excessive biomass growth and even explosion hazards through excessive methane production (Aulenta *et al*., 2009a).

A ground-breaking alternative to this approach is the use of insoluble electrodes to directly and selectively deliver electrons instead of chemicals via microbial electric system (MES) to dechlorinating communities growing as biofilms at the electrode surfaces (Lohner and Tiehm, 2009; Lovley, 2011). A wide diversity of microorganisms is able to convert the chemical energy stored in the chemical bonds of organic compounds to electrical energy through the catalytic reactions under anaerobic conditions (Lovley, 2012). The most important step in MES is the transfer of electrons from bacteria to the electrode (Rabaey *et al*., 2004). During this process, some microorganisms require soluble redox mediators such as methylene blue, viologens, thionines, ferricyanides and quinoid compounds that serve as an electron shuttle between the cells and the electrodes to stimulate the bio-electrochemical conversion process. As an example, Aulenta and colleagues (2007) reported the cessation of TCE dechlorination in the absence of the low-potential redox mediator, methyl viologen. However, TCE degradation resumed when methyl viologen was added. The proposed MES process carries several advantages resulting from the use of electrodes to stimulate biological reduction in the subsurface. Among them are continuous monitoring and direct delivery of electrons to dechlorinating microorganisms in terms of current and potential. In addition, no chemicals are required to be injected, which eliminates the need for transport, storage, dosing and post-treatment (Aulenta *et al*., 2009b).

Reductive dechlorination or dehalogenation (i.e. the substitution of chlorine by a hydrogen atom) is the main pathway used by dechlorinating microorganisms for the stepwise reduction of PCE to TCE, *cis*-DCE (cDCE) and vinyl chloride (VC) before forming the environmentally safe end-product ethene (Futagami *et al*., 2008). A clear understanding of how microbial ecology within MES brings about reductive dechlorination is important for its wider application (Rabaey *et al*., 2004). Pronounced enrichment of microorganisms from *Geobacteraceae*, *Desulfuromonas*, *Desulfitobacteriacea* and *Dehalococcoides* (*Dhc*) groups in mixed consortia have been extensively observed to electrochemically interact with electrodes. This interaction that involves directly donating or accepting electrons from electrode surfaces is exploited in MES to assist in the reductive dechlorination of chlorinated compounds with energy production (Bond *et al*., 2002; Bond and Lovley, 2003; Aulenta *et al*., 2007; 2008; 2009a,b; Strycharz *et al*., 2008). However, a wide diversity of other, as-yet-undefined microorganisms may function in a similar manner. The ability of other dechlorinating populations compared with the mentioned ‘classical’ bacterial groups associated with bio-electrochemical reductive dechlorination has to date been poorly investigated (Lovley, 2012). Hence, we developed a system where MES were fed with PCE-contaminated groundwater consisting of a biostimulated natural microbial population [biostimulation (BS) treatment] and a stimulated population augmented with a dechlorinating consortia *Dhc* strains BAV1, GT and FL2 [BS-bioaugmentation (BS-BA) treatment]. These treatments were compared with control MES with no inoculum or nutrient stimulation. We postulated that it is important to understand the multispecies interactions among the dechlorinating community in order to successfully assess the potential for stimulating the process of decontamination of groundwater. If stimulation of indigenous microbial community can lead to bio-electrochemical PCE transformation, then it could serve as a cost-effective *in situ* remediation practice as it would restrict the need for BA of contaminated subsurfaces. Furthermore, given the recent move in some countries to discourage the use of bio-augmenting agents (Ball, 2013), this approach may reduce the risk of damaging or causing mutation in the natural biome.

The purpose of this study was therefore to identify and evaluate the ability of an indigenous non-*Dhc* dechlorinating community present in PCE-contaminated groundwater that could evolve in MES to accomplish reductive dechlorination along with bioenergy production. In addition, an assessment of the contribution of this indigenous non-*Dhc* dechlorinating population in comparison with classical dechlorinating microorganisms such as *Dhc* was performed. These investigations were carried out using electrochemical analysis and culture-independent polymerase chain reaction (PCR)–denaturing gradient gel electrophoresis (DGGE)-based molecular techniques.

## Results and discussion

### MES-assisted reductive dechlorination of PCE

**Fig. 1:**
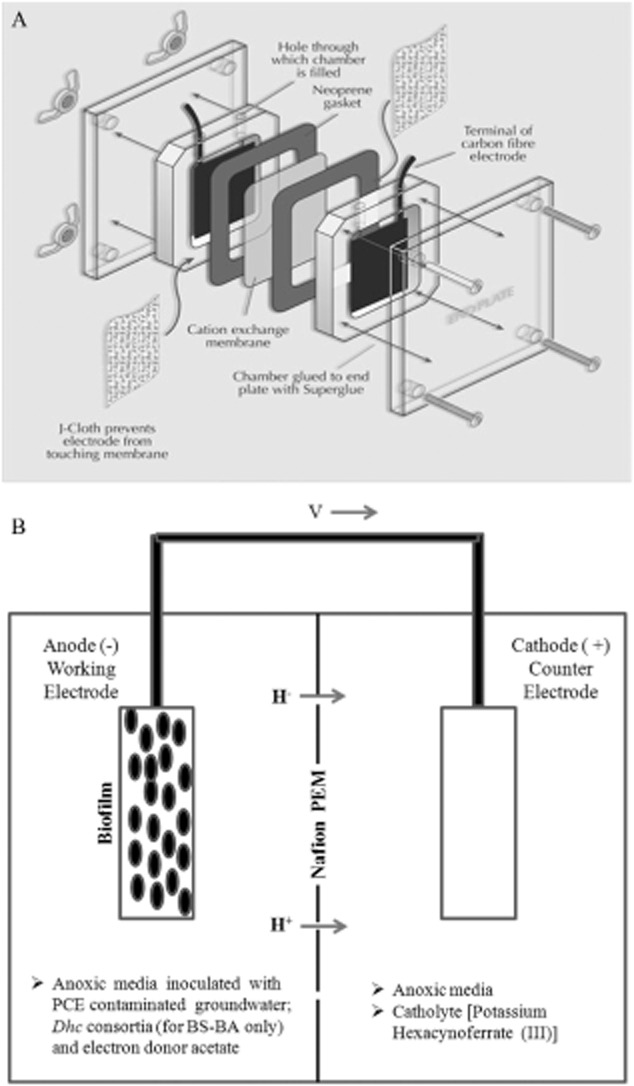
(A) Components of two-chamber NCBE-type MES used in this study (Bennetto, 1990); (B) schematics explaining mechanism of MES.

**Fig. 2:**
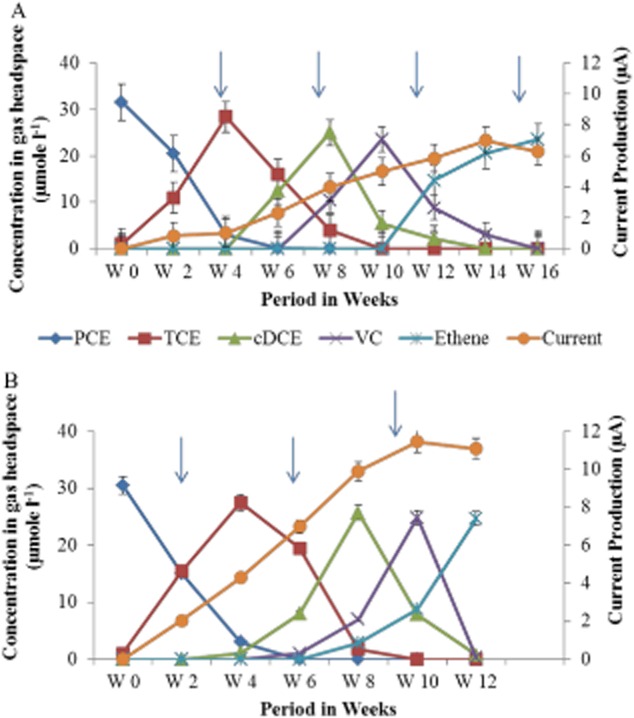
MES-assisted reductive dechlorination of PCE and simultaneous current production in (A) MES 1 and 2 with BS and (B) MES 3 and 4 with BS-BA treatments. Values are average of duplicate cultures. The arrows show when fresh catholyte and electron donors were added.

In this study, we employed a bio-electrochemical system (Fig. 1) to study the microbial reductive dechlorination of PCE under BS and BA regimes. Figure 2 illustrates the cumulative formation of PCE-dechlorinating intermediate products and simultaneous current flow during both BS and BS-BA treatments, when MES were fed with acetate as an electron donor and PCE/electrodes as acceptors. During BS treatment, PCE was completely reduced to ethene over a period of 16 weeks (Fig. 2A). PCE was consumed by week 4, with the subsequent production of TCE. As the TCE concentration was reduced to 15 μmol l^−1^, cDCE was detected by week 6. Daughter products, cDCE and VC co-existed until ethene was formed. In week 16, only after VC was respired did ethene concentration reach its peak. Current production was negligible for first 3 weeks, but as dechlorination progressed, current production increased from week 4 and stabilized between 6.27 and 6.98 μA over the period of 16 weeks of complete dechlorination (Fig. 2A). In contrast, PCE dechlorination did not progress beyond TCE, and current generation was also negligible in the control 1 MES without acetate stimulation (Table 1). These findings showed that BS was beneficial to dechlorination and that the indigenous microbial community (BS) were most likely involved in complete reductive dechlorination given that no *Dhc* were detected in the groundwater samples used for this study. Reductive dechlorination was also accompanied by simultaneous bioenergy production (Fig. 2A).

**Table 1:** Comparative response of BS only and BS-BA treatments against controls during MES assisted PCE dechlorination

Chloroethene	BS only	BS-BA	Control 1 (without acetate)	Control 2 (without acetate and *Dhc* inoculum)	Control 3 (without catholyte)	Control 4 (without electrodes)
PCE	+	+	+	+	+	+
TCE	+	+	+	+	+	+
cDCE	+	+	−	−	−	+
VC	+	+	−	−	−	−
Ethene	+	+	−	−	−	−

+ Presence; − Absence; Control MES (1) medium, electrodes, PCE-contaminated groundwater, catholyte but no acetate; (2) medium, electrodes, PCE-contaminated groundwater, catholyte but no acetate and *Dhc* inoculum; (3) medium, electrodes, PCE-contaminated groundwater, acetate but no catholyte; (4) medium, PCE-contaminated groundwater, acetate, catholyte, but no electrodes.

In BS-BA-treated MES, dechlorination was faster, and the current production was ∼1.6-fold higher than MES run on BS-only treatment (Fig. 2B). PCE dechlorination started immediately, as indicated by the rapid accumulation of TCE by week 2. During BS-BA treatment, PCE was transformed into ethene over 12 weeks (Fig. 2B). Current generation started from week 2 and was stabilized between 11.08 and 11.45 μA by the end of dechlorination in week 12 (Fig. 2B). The *Dhc* and non-*Dhc* communities altogether led to complete PCE dechlorination, where ∼61% of energy production (from Eqn 1 in experimental procedures) was observed to be contributed by non-*Dhc* activities. This indicated the significant role played by non-*Dhc* community in association with the *Dhc* consortia to optimize dechlorination and current output. In contrast, over the experimental period, PCE dechlorination stopped at TCE, and negligible current was observed in control 2 MES, when the *Dhc* microbial culture and acetate were omitted from the poised electrode system (Table 1). This indicates both nutrient stimulation and the electrochemically active *Dhc* bacterial community are important for enhancing the complete reductive dechlorination of PCE to ethene. Interestingly, no methane was produced in the cultures probably because of the presence of mixed culture of a lower number of methanogens compared with dechlorinators. This could prove advantageous for MES as it eliminates the competition for H_2_ between dechlorinating and non-dechlorinating communities.

In both the BS and BS-BA-treated MES, the periodic conversion of the dark yellow catholyte potassium hexacyanoferrate [K_3_Fe(CN)_6_] to pale yellow or colourless resulted in the cessation of current flow and dechlorination. However, replenishment of K_3_Fe(CN)_6_ caused a resumption of current flow and dechlorination, indicating its definitive effect on the electron transfer mechanism of MES. When the catholyte was available as a dissolved compound, the measured rate of PCE dechlorination and current flow was unexpectedly higher from BS-only- and BS-BA-treated MES than that measured in the control 3 MES (Table 1). Wei and colleagues (2012) have also reported K_3_Fe(CN)_6_ as an excellent cathodic electron acceptor for two-chambered MES to obtain high power output. While there are some reports on the benefits of BS or BA, or the combined treatment on PCE dechlorination (Aulenta *et al*., 2007; 2008; 2009a,b), most of these studies have been on samples with *Dhc*, with scant attention being paid to the role of indigenous non- *Dhc* species. The potential roles of these non-*Dhc* species have therefore been comparatively poorly investigated to date. In addition, most studies have been focused on either BS or BA, or BS-BA alone in different systems. Hence, one highlight of this research is that both BS and BS-BA treatments were set up with the same sample under similar experimental conditions allowing for objective comparison of these strategies (unlike in most studies).

### Community analysis

**Fig. 3:**
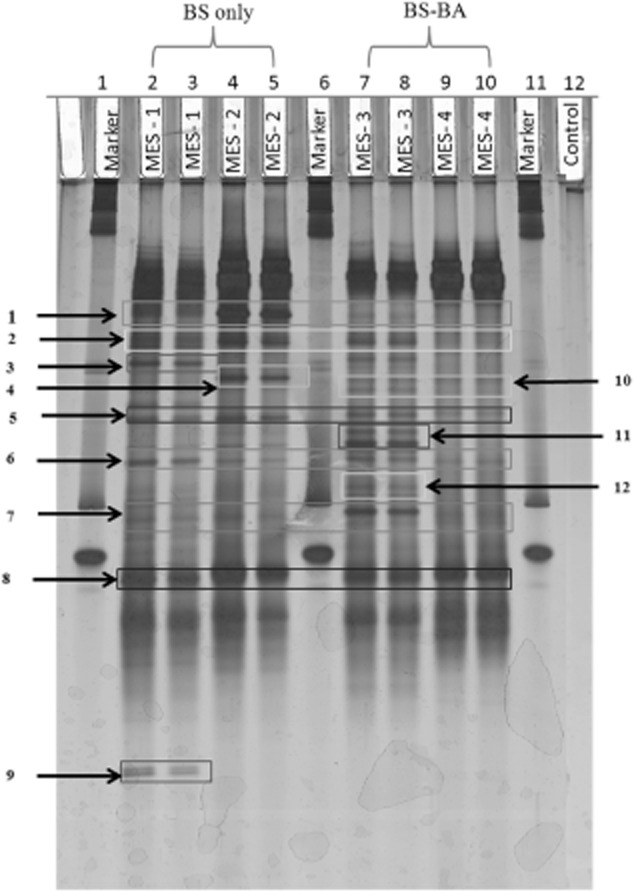
Microbial community fingerprint from MES analysed using DGGE. MES 1 and 2 represent BS only (lanes 2–5); MES 3 and 4 with BS-BA treatment (lanes 7–10), while lane 12 indicates control MES. Lanes 1, 6 and 11 represent marker. Band numbers designate dominant bands excised from DGGE gel for sequence analysis that correspond to the band numbers in Table 2.

Microbial community fingerprints were obtained from DGGE analysis during the MES-assisted reductive dechlorination of PCE from BS-only, BS-BA and control treatments (Fig. 3). No bands were obtained from the control MES (Fig. 3) presumably because of either PCR detection constraints relating to the small volume used for DNA extraction or the absence of nutrient stimulation. Similarly, no amplicons were obtained from archaea-specific PCR indicating the absence or below-detection level of a methanogenic archaea community in the groundwater. No *Dhc* species were detected in PCR-based assays carried out on groundwater samples. To identify the likely electrochemically active bacterial species carrying out reductive dechlorination in BS and BS-BA MES, the dominant bands (operational taxonomic units) based on strong band intensity were excised from the DGGE gel and sequenced. Sequencing results indicated significant phylogenetic diversity in the species identified. Microbial community belonging to facultative anaerobic bacteria including the taxa *Spirochaetes*, *Firmicutes*, γ-*Proteobacteria*, δ-*Proteobacteria* and *Bacteroidetes* were detected in both BS-only- and BS-BA-treated MES, while *Chloroflexi* was detected only in BS-BA MES (Table 2).

**Table 2:** Overview of the bacterial species identified based on the occurrence of a dominant DGGE pattern obtained from MES-assisted PCE dechlorination

Excised DGGE Bands	Accession No.	Closest matches overall (NCBI database)	Maximum % similarity	Phylum	Detected within (treatment)
1	AF349763.2	Uncultured bacterium DCE33 16S ribosomal RNA gene, partial sequence	97	Spirochaetes	BS and BS-BA
2	AY667253.1	Uncultured bacterium clone TANB18 16S ribosomal RNA gene, partial sequence	96	Spirochaetes	BS and BS-BA
3	GQ377125.1	Bacterium enrichment culture clone DPF05 16S ribosomal RNA gene, partial sequence	96	Spirochaetes	BS and BS-BA
4	AF357916.2	*Spirochaeta* sp. 16S ribosomal RNA gene, partial sequence	98	Spirochaetes	BS and BS-BA
5	AJ249227.1	Bacterium DCE25 16S rRNA gene	96	Firmicutes	BS and BS-BA
6	JF920024.1	*Enterobacter* sp. 16S ribosomal RNA gene, partial sequence	98	γ-Proteobacteria	BS and BS-BA
7	JF689075.1	Bacterium enrichment culture clone ALO1_GLFRUDD03F0MQ1 16S ribosomal RNA gene, partial sequence	96	γ-Proteobacteria	BS and BS-BA
8	DQ903931	*Desulfovibrio* Sp. GmS2 (SRB enrichment clone) 16S ribosomal RNA gene, partial sequence	97	δ-Proteobacteria	BS and BS-BA
9	HM488066.1	Uncultured bacterium clone ZM4-54 16S ribosomal RNA gene, partial sequence	97	Bacteroidetes	BS and BS-BA
10	AY165308.1	*Dehalococcoides* sp BAV1 16S rRNA gene, partial sequence	100	Chloroflexi	BS-BA only
11	AY914178.1	*Dehalococcoides* sp GT, 16S rRNA, partial sequence	100	Chloroflexi	BS-BA only
12	AF357918.2	*Dehalococcoides* sp FL2, 16S rRNA gene, partial sequence	100	Chloroflexi	BS-BA only

#### Community detected within BS-only-treated MES

In addition to the non-detection of *Dhc* in the original groundwater samples, no *Dhc* amplicons were obtained from optimized PCR assays during the dechlorination process, within BS-only treated MES. This indicated either the absence of *Dhc* species or their presence below PCR detection limits and possible ecological insignificance in groundwater sample. However, the *Spirochaetes* showed 96–98% similarity to uncultured bacterial clones DCE33, TANB18, DPF05 and *Spirochaeta* sp. (Table 2). DGGE bands that showed 96% similarity with an unidentified bacterial clone DCE25 was grouped under *Firmicutes*, while bands putatively assigned to the phyla γ-*Proteobacteria* showed 98% and 96% sequence similarity to *Enterobacter* species and the bacterial clone ALO1_GLFRUDD03F0MQ1, respectively. Other bands showed 97% similarity to the well-known dechlorinator *Desulfovibrio* species under the taxonomic group of δ-*Proteobacteria* and uncultured bacterial clones ZM4-54 within the phylum *Bacteroidetes*.

The detection of *Spirochaetes* group in this study is not unusual as they have also been reported in other PCE-reducing cultures inoculated with a sample from a chloroethene-contaminated site (Gu *et al*., 2004; Macbeth *et al*., 2004; Dong *et al*., 2011). *Spirochaetes* either utilize H_2_ or ferment carbohydrates and other complex substrates to acetate and other substances that are utilized during organohalide respiration. Under the γ-*Proteobacteria*, clone ALO1_GLFRUDD03F0MQ1 is a known 1,2dichloroethane dechlorinator (Low *et al*., 2011), while *Enterobacter* species are the only facultative anaerobes reported so far to reductively dechlorinate PCE to cDCE (Holliger *et al*., 1999). Studies by Löffler and colleagues (2003), and Sun and colleagues (2000) have shown the contribution of a marine dechlorinating *Desulfivibrio* species in the reductive dechlorination that forms syntrophic associations with other dechlorinating bacteria to produce hydrogen by the transformation of organic compounds added to the medium. The hydrogen produced can then be transferred to dehalogenating bacteria and thus support microbially mediated reductive dechlorination (Drzyzga *et al*., 2001; Eydal *et al*., 2009). Bacterial clones ZM4-54 under the *Bacteriodetes* may, through fermentation, supply small organic molecules or H_2_ necessary for the growth of dechlorinating bacteria (Tancsics *et al*., 2010); however, the metabolic function of this organism is still unclear. The bacterium DCE25 in TCE and cDCE cultures acts as an acetate-fermenting organisms providing energy and a carbon source for the dechlorinating microbes (Flynn *et al*., 2000).

#### Community detected within BS-BA-treated MES

In addition to the earlier H_2_-utilizing bacterial communities, only in MES run with BS-BA treatment augmented with *Dhc* strains BAV1, GT and FL2 were the *Chloroflexi* phyla detected (Table 2). The *Dhc* strains GT, FL2 and BAV1 have previously been well documented to cometabolically transform PCE to ethene (Futagami *et al*., 2008).

The faster dechlorination and almost twofold increase in current flow in BS-BA-treated MES when compared with the BS-only MES could be due to synergistic activity between the dechlorinating *Dhc* and non-*Dhc* species. Members of the indigenous non-*Dhc* community were believed to have contributed significantly to electron transfer as calculations using Eqn 1 showed that they could have been responsible for ∼61% of the total current generated within BS-BA-treated MES. In spite of the absence of *Dhc* strains, BS-MES cultures containing an indigenous non-*Dhc* community were equally capable of completely reducing PCE. Overall, these comparative treatments highlighted the potential of mixed non-*Dhc* bacterial communities evolved in MES, which most likely contributed to the electron transfer mechanism supporting complete reductive dechlorination of PCE with and without *Dhc*. A study by Aulenta *et al*. (2009b) also reported the key role played by β-, δ- and γ-*Proteobacteria*, and *Firmicutes* besides *Dhc* in a mixed culture carrying out the dechlorination of TCE to non-chlorinated end-products within MES. Although the dechlorinating capabilities of these detected indigenous non-*Dhc* population have been well-studied (Gu *et al*., 2004; Macbeth *et al*., 2004; Dong *et al*., 2011), knowledge about their electrochemical properties is currently limited.

### Anode biofilms

**Fig. 4:**
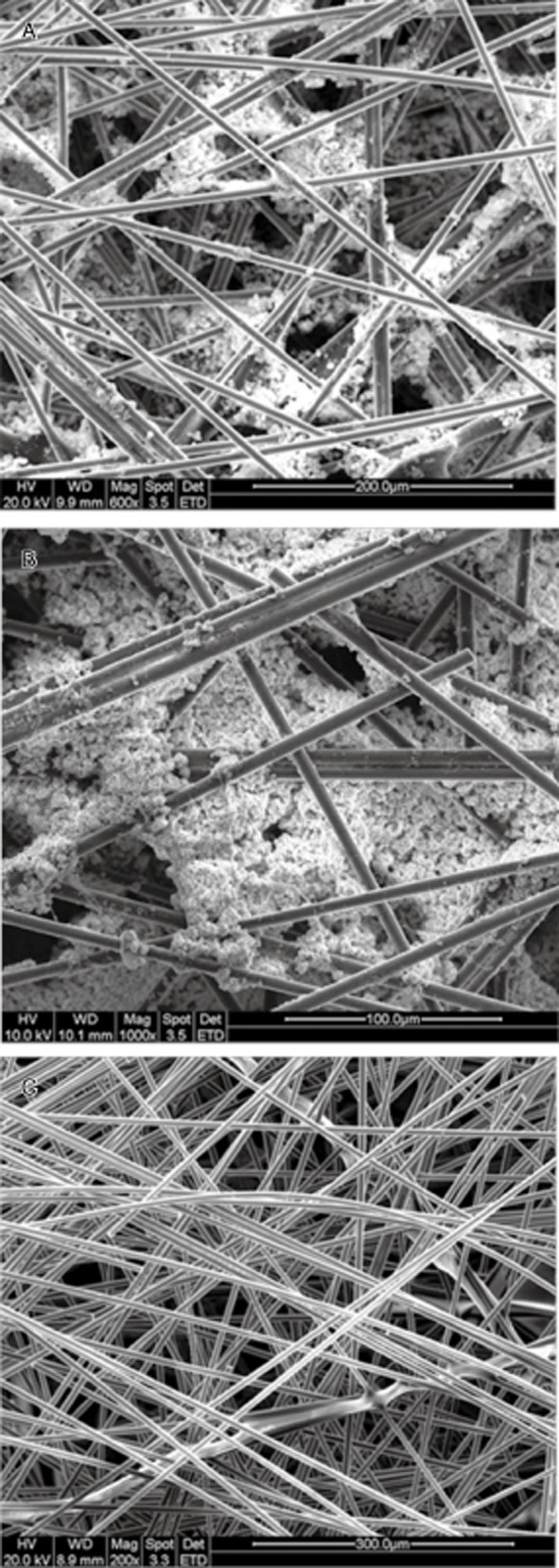
Environmental scanning electron photomicrographs of bacterial biofilms grown on carbon fibre anode surfaces during PCE dechlorination within (A) MES with BS only (B) MES with BS-BA and (C) control 1–3 MES at the end of experiment. No biofilm was observed in controls 1–3 MES; however, thick biofilm was noticeable in MES run with BS and BS-BA treatments.

The analysis of the anode electrode surfaces via environmental scanning electron microscope (ESEM) conducted at the end of the experiment revealed that the surfaces of carbon fibre electrodes in both BS-only- and BS-BA-treated MES had been colonized (Fig. 4A and B). In contrast, no bacterial cells were observed at the anode surface of the controls 1–3 MES (Fig. 4C) that could be due to the lack of nutrient stimulation and/or biologically active microorganisms. This could have led to incomplete PCE degradation observed in these control samples unlike in BS-only and BS-BA samples. Mixed bacterial culture in BS-BA-treated MES formed complex cellular aggregates compared with sparsely distributed cells in BS-only MES (Fig. 4A and B). Microbial cells did not support complete dechlorination and energy production when the supply of electrons to the electrode was discontinued in control 4 MES (Table 1). Overall, this result demonstrated that the bacterial community present in BS-only and BS-BA sets of MES could bio-electrochemically interact with electrodes as electron acceptors by forming a stable, attached population that can produce electrical current via reductive dechlorination coupled to electron transfer to the electrodes.

### Reductive dechlorination and bacterial electrochemical activity

In this study, DGGE band sequencing yielded sequences similar to those of several previously described H_2_-oxidizing *Dhc* and non-*Dhc* bacteria. These findings suggest that facultative anaerobic bacterial species are capable of growing in MES using the electrode as an acceptor, further indicating their electrochemical potential or at least redox controlling properties. MES enhances the growth of bacteria that can use the electrode as an electrode acceptor as bacteria have been observed to gain more energy when using an electrode as an electron acceptor than when they use protons (Rabaey *et al*., 2004). In recent years, it has been demonstrated that a direct electron transfer between bacteria and electrodes is possible within MES (Strycharz *et al*., 2008). This study seems to indicate that a similar approach might have pursued by non-*Dhc* bacteria especially with regards to ethene formation that strive to access electrons throughout PCE dechlorination that was utilized for energy production.

As previous reports on this topic have been limited to a few microbes (Lovley, 2012), this study focused on other organisms to elucidate their electrochemical mechanism during PCE remediation. We report here that non-*Dhc* microorganisms within MES supplied with acetate as an electron donor and PCE/electrode as acceptors seemed to play a role in the complete PCE dechlorination with current production. However, to implement this strategy competitively with BS-BA, a clear understanding about electron transfer mechanisms between non-*Dhc* species and electrodes with nutrient stimulation is necessary to optimize the dechlorination rates and current output. In order to scale-up this strategy for successful *in situ* application, extensive testings based on subsurface characteristics and site-specific design needs to be studied. Williams and colleagues (2010) for the first time demonstrated the *in situ* applicability of graphite electrodes in the subsurface serving as electron acceptors for microbial stimulation during uranium bioremediation at Rifle site in Colorado. A similar approach may be employed for the remediation of chlorinated solvents. Preliminary investigations of environmental factors and complex microbial interactions at contaminated sites will decide the potential of MES for PCE bioremediation. If a native dechlorinating community was found to be capable of self-mediated electrochemical conversion of PCE, it would further eliminate the need of bio-augmenting the subsurface that will eventually reduce remediation cost. Future research necessitates investigating the possibility of electrode-dependent, microbially catalysed PCE degradation where non-*Dhc* bacteria can utilize the electrode as the sole electron donor. Altogether, this strategy could prove advantageous, especially where electron donor delivery to subsurface has always been a challenge. Nevertheless, it will be of fundamental importance to focus on mechanisms involved in the extracellular electron transfer process between microorganism and the electrodes to develop strategies to maximize dechlorination rates.

To conclude, this study highlighted the electrochemical potential of indigenous non-*Dhc* dechlorinators compared with *Dhc* species during the complete dechlorination of PCE to ethene via MES. Although the direct involvement of mixed *Dhc* culture in the electron transfer process was expected in BS-BA run MES, the potential of non-*Dhc* dechlorinators exhibiting similar mechanisms within BS-MES was observed. Microbial communities in the MES evolved specifically as an optimized biocatalyser generating a stable power output, opening a perspective for the development of a new sustainable bioremediation strategy. Clearly, research is needed to further elucidate the electrochemical mechanism of these as-yet-undefined non-*Dhc* dechlorinators in order to advance this field in a rational manner.

## Experimental procedures

### Materials

All chlorinated ethenes, ethene and other chemicals including potassium hexacyanoferrate (III), sulphuric acid and hydrogen peroxide required for the experimental setup and analytical measurements were purchased from Sigma-Aldrich (Sydney, NSW, Australia) with a minimum purity of 99.5%. All gases were ordered from Coregas (Melbourne, Vic., Australia).

### Groundwater sample collection

For this study, we selected a chloroethene contaminated site located in Victoria, Australia. A sample of contaminated groundwater (4 l) with a PCE concentration of 130 μg l^−1^ was collected from the monitoring well as per the protocol suggested by Ritalahti *et al*. (2010). A flow-through cell (YSI, Melbourne, Vic., Australia) recorded pH, oxidation-reduction potential, specific conductance, temperature, dissolved oxygen and turbidity of groundwater. When geochemical parameters were stabilized, the flow-through cell was disconnected and replicate samples collected consecutively without flow interruption. Sample containers consisted of sterile and N_2_^–^ purged high-density polyethylene Nalgene 4 l bottles with polypropylene screw caps (Thermo Fisher Scientific Australia, Sydney, NSW, Australia). During sampling, bottles were filled to capacity and stored on ice that was then express-shipped to the analytical laboratory. Upon arrival, samples were placed in the dark at 4°C.

### Media preparation

An anoxic PCE-dechlorinating mineral media was enriched and maintained as per the guidelines presented by Löffler and colleagues (2005) and the American Type Culture Collection (ATCC; http://www.atcc.org). Media was prepared in Wheaton serum bottles of 125 ml nominal volume containing 75 ml of growth medium and 20 ml of PCE-contaminated groundwater as an inoculum amended with 5 mM acetate as an electron donor and 5 μl of PCE as an electron acceptor. The bottles were sealed with Teflon-coated butyl rubber septa and aluminium crimp caps (Alltech, Melbourne, Vic., Australia). Hydrogen (5% in 95% nitrogen) was added in the headspace (5–10% of the headspace volume of a bottle) at a low partial pressure of 9 kPa. Cultures were prepared under strict anaerobic conditions maintained in an anaerobic glove box (La-Petite, Thermo Fisher Scientific Australia) using N_2_ : CO_2_ at the ratio of 80%:20%. Resazurin redox indicator was added to the groundwater to denote reduced conditions. Immediately upon setup, media turned clear from pink tint (given by the resazurin redox indicator added to the groundwater) indicating establishment of reduced conditions.

### MES construction and operation

For this study, we employed typical two-chamber NCBE-type MES (National Centre for Biotechnology Education, Reading, UK). The MES chamber (7.5 × 9.0 × 5.5 cm) consisted two electrode compartments (60 × 70 × 10 mm; 10 ml each) separated by a reinforced Nafion^424^ proton exchange membrane (PEM) 0.007″ thickness (Sigma-Aldrich) (Fig. 1A; Bennetto, 1990). Compartments were kept watertight by placing rubber gaskets between chambers and by bolting two Perspex sheets together. The PEM was pretreated by boiling in H_2_O_2_ (30%), then in 0.5 M H_2_SO_4_ and finally in de-ionized (DI) water, each for 1 h, and then stored in DI water prior to being used. The carbon fibre electrodes (3.2 × 4 cm) were soaked in DI water prior to use (Aulenta *et al*., 2007). Sampling ports were sealed with rubber stoppers, while carbon electrodes were attached to copper wires fed through rubber stoppers in the sampling port.

Two out of the four MES were run on a BS-only approach where MES were fed with groundwater comprising an indigenous microbial population. The remaining two MES were dedicated to the BS-BA treatment where the same groundwater was amended with a dechlorinating mixed consortia of *Dhc* species FL2 (*Dehalococcoides* sp. ATCC® BAA-2098™), BAV1 (*Dehalococcoides* sp. ATCC BAA-2100™) and GT (*Dehalococcoides* sp. ATCC BAA-2099™) outsourced from ATCC (http://www.atcc.org). MES were established by supplying acetate as electron donor and PCE/electrodes as acceptors. Ten millilitres of anoxic media inoculated with PCE-contaminated groundwater and acetate was transferred anaerobically from 125 ml Wheaton bottles into the working electrode chambers of all four MES using a gas-tight syringe. In parallel, the counter electrode was filled with an equal volume of a reduced mineral media and was spiked with 20 mM potassium hexacyanoferrate (III) [K_3_Fe(CN)_6,_ catholyte] in phosphate buffer (Fig. 1B). The *Dhc* mixed consortia (1 × 10^3^ cells ml^−1^ each) was injected only into the working electrode compartment of MES 3 and 4. Chambers were then flushed with 80:20 N_2_ : CO_2_ mixed gases. The whole process was carried out in an anaerobic chamber under strict anoxic conditions. Once sealed, MES were maintained at 22–25°C in the dark under gentle magnetic stirring in an attempt to promote the growth and adhesion of dechlorinating bacteria on the surface of carbon fibre electrodes. The working electrode was poised at −450 mV (versus standard hydrogen electrode). Electrochemical measurements were taken using a Fluke 289 digital true RMS multimeter (RS Components, Melbourne, Vic., Australia). Cells were monitored over 16 weeks (112 days) with current and voltage being recorded throughout the dechlorination process. The energy production (%) from BS and BS-BA treatments was calculated as follows:


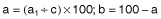
(1)

where a_1_ = current from BS, a = % current from BS, b = % current from BA, and c = total current from BS-BA. The experiment was followed by multiple control MES with: (i) medium, electrodes, PCE-contaminated groundwater, catholyte but no acetate; (ii) medium, electrodes, PCE-contaminated groundwater, catholyte but no acetate and *Dhc* inoculum; (iii) medium, electrodes, PCE-contaminated groundwater, acetate but no catholyte; (iv) medium, PCE-contaminated groundwater, acetate, catholyte, but no electrodes.

### Microbial community analysis

Genomic DNA extraction from groundwater samples treated with MES was carried out as described by Löffler and colleagues (2005) and 16S rRNA gene fragments were amplified with bacterial universal primer set 341 F-GC/518R (Muyzer *et al*., 1993) to detect the presence of indigenous and likely dechlorinating bacterial communities. The *Archaea*-specific primers A109f and A934b were also used for detection of methanogens under domain *Archaea* as described by Høj *et al*. (2008). *Dhc*-specific primers 1F-GC and 259R with *Dhc* strain GT, FL2 and BAV1 (positive controls) were used for the detection of *Dhc* in groundwater samples using touchdown PCR that was optimized as described by Kim and colleagues (2010). Amplified 200 bp PCR fragments were further analysed by DGGE as described by Patil and colleagues (1993). Dominant DGGE bands were excised using sterile razor blades and incubated in two volumes of DNA elution buffer (0.5 mmol l^−1^ ammonium acetate, 10 mmol l^−1^ magnesium acetate, 1 mmol l^−1^ ethylenediamine tetraacetic acid pH 8 and 0.1% sodium dodecyl sulfate) overnight at 37°C. DNA was then precipitated with two volumes of absolute ethanol, air-dried, resuspended in 20 μl nuclease-free water and stored at −20°C until re-amplification (McKew *et al*., 2007). Re-amplification was performed using 341F/518R primers. Re-amplified PCR products were purified using the Wizard® SV gel and PCR clean up system (Promega, Madison, WI, USA) as per the manufacturer's instructions. The eluted DNA was checked for concentration and purity using a Nanodrop Lite spectrophotometer (Thermo Scientific Australia). Samples were then sent to the Australian Genome Research Facility for sequencing using an automated sequencer, ABI 3730. Nucleotide sequences were analysed using SEQUENCHER software (Sequencher Version 4.1.4, GeneCodes Corp., Ann Arbor, MI, USA), and homology searches were completed with the BLAST server of the National Centre for Biotechnology Information (NCBI) using a BLAST algorithm (http://www.ncbi.nlm.gov.library.vu.edu.au/BLAST/) for the comparison of a nucleotide query sequence against a nucleotide sequence database (blastn).

### Analytical procedures

Every 2 weeks, 50 μl chlorinated ethenes were removed from the gas headspace of both working and counter electrode compartments using a gas tight, sample-lock Hamilton syringe (Alltech) and analysed by an HP 6890 gas chromatographic (GC) system equipped with a 5973 mass spectrometry and flame ionizing detector and a Porabond-Q column (0.32 mm by 25 m) (Agilent Tech, Melbourne, Vic., Australia). The GC settings were: injector temperature 200°C; detector temperature 300°C; oven temperature 3 min at 40°C, followed by an increase of 10°C min^−1^ to 70°C, followed by an increase of 15°C min^−1^ to 250°C for 7 min; and carrier gas (He) with a flow rate of 2 ml min^−1^. External standards at six different concentrations from 0 to 30 μM were used for calibration. Electron donors were replenished every time analyses indicated they were exhausted.

### Microscopy

At the end of the experiment, anodes from all MES were removed, cut into small pieces using a sterile razor blade and washed with phosphate buffer (pH 7.0) to remove loosely attached cells. Subsequently, samples were observed using a Quanta 200 ESEM (FEI Company, Melbourne, Vic., Australia). The ESEM was operated at 10–20 kV, and images were captured digitally.

### Nucleotide sequence accession numbers

All bacterial sequences have been deposited in the NCBI database under accession numbers JX495100–JX495111.

## References

[b1] ATSDR (2007). http://www.Atsdr.cdc.gov.

[b2] Aulenta F, Catervi A, Majone M, Panero S, Reale P, Rossetti S (2007). Electron transfer from a solid-state electrode assisted by methyl viologen sustains efficient microbial reductive dechlorination of TCE. Environ Sci Technol.

[b3] Aulenta F, Canosa A, Majone M, Panero S, Reale P, Rossetti S (2008). Trichloroethene dechlorination and H_2_ evolution are alternative biological pathways of electric charge utilization by a dechlorinating culture in a bio-electrochemical system. Environ Sci Technol.

[b4] Aulenta F, Canosa A, Roma LD, Reale P, Panero S, Rossetti S, Majone M (2009a). Influence of mediator immobilization on the electrochemically assisted microbial dechlorination of trichloroethene (TCE) and *cis*- dichloroethene (*cis*-DCE). J Chem Technol Biotechnol.

[b5] Aulenta F, Canosa A, Reale P, Rossetti S, Panero S, Majone M (2009b). Microbial reductive dechlorination of trichloroethene to ethene with electrodes serving as electron donors without the external addition of redox mediators. Biotechnol Bioeng.

[b6] Ball AS (2013).

[b7] Bennetto HP (1990). Electricity generation by micro-organisms. Biotechnol Ed.

[b9] Bond DR, Lovley DR (2003). Electricity production by *Geobacter sulfurreducens* attached to electrodes. Appl Environ Microbiol.

[b8] Bond DR, Holmes DE, Tender LM, Lovley DR (2002). Electrode–reducing microorganisms that harvest energy from marine sediments. Science.

[b10] Dong Y, Butler EC, Philp RP, Krumholz LR (2011). Impacts of microbial community composition on isotope fractionation during reductive dechlorination of tetrachloroethylene. Biodegradation.

[b11] Drzyzga O, Gerritse J, Dijk JA, Elissen H, Gottschal JC (2001). Coexistence of a sulphate reducing *Desulfovibrio* species and the dehalorespiring *Desulfitobacterium frappieri* TCE1 in defined chemostat cultures grown with various combinations of sulfate and tetrachloroethene. Environ Microbiol.

[b12] Eydal HS, Jagevall S, Hermansson M, Pedersen K (2009). Bacteriophage lytic to *Desulfovibrio aespoeensis* isolated from deep groundwater. ISME J.

[b13] Flynn SJ, Löffler FE, Tiedje JM (2000). Microbial community changes associated with a shift from reductive dechlorination of PCE to reductive dechlorination of *cis*-DCE and VC. Environ Sci Technol.

[b14] Futagami T, Goto M, Furukawa K (2008). Biochemical and genetic bases of dehalorespiration. Chem Record.

[b15] Gu AZ, Hedlund BP, Staley JT, Strand SE, Stensel HD (2004). Analysis and comparison of the microbial community structures of two enrichment cultures capable of reductively dechlorinating TCE and cis-DCE. Environ Microbiol.

[b16] Høj L, Olsen RA, Torsvik VL (2008). Effects of temperature on the diversity and community structure of known methanogenic groups and other archaea in high arctic peat. ISME J.

[b17] Holliger C, Wohlfarth G, Diekert G (1999). Reductive dechlorination in the energy metabolism of anaerobic bacteria. FEMS Microbiol Rev.

[b18] Kim BH, Baek KH, Cho DH, Sung Y, Koh SC, Ahn CY (2010). Complete reductive dechlorination of tetrachloroethene to ethene by anaerobic microbial enrichment culture developed from sediment. Biotechnol Lett.

[b19] Löffler FE, Cole JR, Ritalahti KM, Tiedje JM, Haggblom MM, Bossert ID (2003). Diversity of dechlorinating bacteria. Dehalogenation: Microbial Processes and Environmental Applications.

[b20] Löffler FE, Sanford RA, Ritalahti KM (2005). Enrichment, cultivation and detection of reductively dechlorinating bacteria. Meth Enzymol.

[b21] Lohner ST, Tiehm A (2009). Application of electrolysis to stimulate microbial reductive PCE dechlorination and oxidative VC biodegradation. Environ Sci Technol.

[b22] Lovley DR (2011). Powering microbes with electricity: direct electron transfer from electrodes to microbes. Environ Microbiol Rep.

[b23] Lovley DR (2012). Electromicrobiology. Annu Rev Microbiol.

[b24] Low A, Zemb O, Manefield M (2011). Characterisation of a Unique Mixed Bacteria Culture that Degrades 1,2-Dichloroethane in Low pH Conditions.

[b25] Macbeth TW, Cummings DE, Spring S, Petzke LM, Sorenson KS (2004). Molecular characterization of a dechlorinating community resulting from in situ biostimulation in a trichloroethene-contaminated deep, fractured basalt aquifer and comparison to a derivative laboratory culture. Appl Environ Microbiol.

[b26] McKew BA, Coulon F, Osborn AM, McGenity TJ, Timmis KN (2007). Effects of temperature and biostimulation on oil-degrading microbial communities in temperate estuarine waters. Environ Microbiol.

[b27] Moran MJ, Zogorski JS, Squillace PJ (2007). Chlorinated solvents in groundwater of United States. Environ Sci Technol.

[b28] Muyzer G, de Waal EC, Uitterlinden A (1993). Profiling of complex microbial populations using denaturing gradient gel electrophoresis analysis of polymerase chain reaction- amplified genes coding for 16S rRNA. Appl Environ Microbiol.

[b29] Patil SS, Kumar MS, Ball AS (2010). Microbial community dynamics in anaerobic bioreactors and algal tanks treating piggery wastewater. Appl Microbiol Biotechnol.

[b30] Rabaey K, Boon N, Siciliano SD, Verhaege M, Verstraete W (2004). Biofuel cells select for microbial consortia that self-mediate electron transfer. Appl Environ Microbiol.

[b31] Ritalahti KM, Hatt JK, Petrovskis E, Löffler FE, Timmis KN (2010). Groundwater sampling for nucleic acid biomarker analysis. Handbook of Hydrocarbon and Lipid Microbiology.

[b32] Strycharz SM, Woodad TL, Johnson JP, Nevin KP, Saford RA, Löffler FE, Lovely DR (2008). Graphic electrode as sole electron donor for reductive dechlorination of tetrachloroethene by *Geobacter loveleyi*. Appl Environ Microbiol.

[b33] Sun B, Cole JR, Sanford RA, Tiedje JM (2000). Isolation and characterization of *Desulfovibrio dechloracetivorans* sp. nov., a marine dechlorinating bacterium growing by coupling the oxidation of acetate to the reductive dechlorination of 2-chlorophenol. Appl Environ Microbiol.

[b34] Tancsics A, Szabo I, Baka E, Szoboszlay S, Kukolya J, Kriszt B, Marialigeti K (2010). Investigation of catechol 2,3-dioxygenase and 16S rRNA gene diversity in hypoxic, petroleum hydrocarbon contaminated groundwater. Syst Appl Microbiol.

[b35] Wei L, Han H, Shen J (2012). Effects of cathodic electron acceptors and potassium ferricyanide concentrations on the performance of microbial fuel cell. Int J Hydrogen Energy.

[b36] Williams KH, Nevin KP, Franks A, Englert A, Long PE, Lovley DR (2010). Electrode-based approach for monitoring in situ microbial activity during subsurface bioremediation. Environ Sci Technol.

